# Zebrafish *foxo3b* Negatively Regulates Canonical Wnt Signaling to Affect Early Embryogenesis

**DOI:** 10.1371/journal.pone.0024469

**Published:** 2011-09-07

**Authors:** Xun-wei Xie, Jing-Xia Liu, Bo Hu, Wuhan Xiao

**Affiliations:** Key Laboratory of Aquatic Biodiversity and Conservation, Institute of Hydrobiology, Chinese Academy of Sciences, Wuhan, People's Republic of China; Hong Kong University of Science and Technology, China

## Abstract

FOXO genes are involved in many aspects of development and vascular homeostasis by regulating cell apoptosis, proliferation, and the control of oxidative stress. In addition, FOXO genes have been showed to inhibit Wnt/β-catenin signaling by competing with T cell factor to bind to β-catenin. However, how important of this inhibition *in vivo*, particularly in embryogenesis is still unknown. To demonstrate the roles of FOXO genes in embryogenesis will help us to further understand their relevant physiological functions. Zebrafish *foxo3b* gene, an orthologue of mammalian *FOXO3*, was expressed maternally and distributed ubiquitously during early embryogenesis and later restricted to brain. After morpholino-mediated knockdown of *foxo3b*, the zebrafish embryos exhibited defects in axis and neuroectoderm formation, suggesting its critical role in early embryogenesis. The embryo-developmental marker gene staining at different stages, phenotype analysis and rescue assays revealed that *foxo3b* acted its role through negatively regulating both maternal and zygotic Wnt/β-catenin signaling. Moreover, we found that foxo3b could interact with zebrafish β-catenin1 and β-catenin2 to suppress their transactivation *in vitro* and *in vivo*, further confirming its role relevant to the inhibition of Wnt/β-catenin signaling. Taken together, we revealed that *foxo3b* played a very important role in embryogenesis and negatively regulated maternal and zygotic Wnt/β-catenin signaling by directly interacting with both β-catenin1 and β-catenin2. Our studies provide an *in vivo* model for illustrating function of FOXO transcription factors in embryogenesis.

## Introduction

Forkhead box O (FOXO) transcription factors, homologues of DAF-16 (the *Caenorhabditis elegans* ortholog), including *FOXO1, FOXO3a, FOXO4* and *FOXO6* in mammalian, are important effectors in insulin/PI3K/Akt signaling pathway. Akt (also known as protein kinase B) can phosphorylate FOXO proteins at three conserved residues upon growth factors stimulation, and result in nuclear exclusion of FOXO proteins, thereby inhibiting FOXO-dependent transcription [1]. On the contrary, increased cellular oxidative stress localizes FOXO proteins to the nucleus, where FOXO proteins bind as monomers to their cognate DNA targeting sequences.

FOXO proteins function as master signaling integrators and participate in a series of dynamic gene expression programs upon various environmental stimuli. FOXO genes are reported to be involved in the regulation of apoptosis, cell cycle progression, and control of oxidative stress, DNA damage repair and cellular differentiation [2]. Most of these environmental stimuli result in post-translational modifications of FOXO proteins, which include but are not limited to phosphorylation, ubiquitylation and acetylation, and affect nuclear/cytoplasmic trafficking of FOXO proteins [1].

Knockout of *FOXO1* in mice causes embryonic lethality due to vascular defects, while knockout of *FOXO3a* only renders the FOXO3a^-/-^ female mice to have an age-dependent reduced fertility and knockout of *FOXO4* does not have an obvious phenotype [3]. These observations suggest that *FOXO* genes have quite diverse physiological roles during vertebrate embryogenesis. However, the underling mechanisms are still not well defined.

Wnt/β-catenin signaling has been revealed participating in the formation of the vertebrate embryonic axes and neuroectoderm during embryogenesis [4]. The role of Wnt/β-catenin signaling during embryogenesis has been well characterized by zebrafish model. In zebrafish, maternally Wnt/β-catenin signaling is essential for the formation of organizer (also known as “shield”). But zygotic Wnt/β-catenin signaling is activated by Wnt ligands after MBT (mid-blastula transition) to antagonize the organizer and be involved in anterior-posterior patterning of the neural axis. Zebrafish embryos homozygous for mutation in the *wnt8* locus show significant expansion of the shield and almost absent expression of ventro-lateral mesoderm markers [5], similar to the phenotype observed in morpholino-mediated *wnt8* knockdown morphants. These data demonstrate that *wnt8* functions to promote the ventro-lateral fate and antagonize the organizer. In addition, the repression of organizer by *wnt8* is mediated by Wnt direct target genes, including *ved*, *vent* and *vox* [6], [7]. During the establishment of rostral-caudal compartments of the vertebrate neural tube, exaggerated Wnt signaling leads to loss of rostral neural domains [7]. Over-expression of *wnt8* in zebrafish leads to changes in expression of its target genes and results in anterior defects [8], while *wnt8* mutants and *wnt8* morphants display expanded forebrain marker expression. Wnt inhibitors, such as *cerberus*, *frzb1* and *dickkopf1*, all function as head inducers [9], [10], [11]. Moreover, as a DNA binding factor in β-catenin transcriptional complex, *tcf3* is essential for forebrain formation by repressing the caudal genes induced by Wnt ligands in zebrafish [12], [13]. Its dominant negative form, dn*TCF*, lacking the β-catenin binding domain and acting exclusively as a repressor, can efficiently promote anterior nervous system [12], and induce the late ectopic expression of dorsal-specific genes in marginal region [14]. In addition, its N-terminal β-catenin–binding domain (tcfBD) is its another dominant negative construct, which inhibits Wnt/β-catenin signaling by depleting the functional β-catenin protein pool. In embryos with ectopic expression of tcfBD, most embryos display ectopic dorsal-specific genes expression and low ventral marker gene *eve1* (even-skipped 1) expression [14].

Interestingly, it has been reported that β-catenin can directly bind to FOXO and enhance FOXO transcription activity [15]. On the contrary, FOXO competes with TCF for interaction with β-catenin, thereby inhibiting TCF transcriptional activity [16]. These facts raise the possibility that FOXO might affect vertebrate embryogenesis through inhibiting Wnt/β-catenin signaling.

While investigating the function of zebrafish *eaf1/2* during embryogenesis, we identified *foxo3b* [initially named as *zFKHR/foxO5* [17], now named as *foxo3b* in ZFIN] as one of strongly suppressed genes [18]. *Foxo3b* is an orthologue of mammalian *FOXO3*. To enrich our understanding about the function of vertebrate FOXO genes during embryogenesis, we are interested in figuring out the role of *foxo3b* during early embryogenesis by taking advantage of zebrafish model.

## Materials and Methods

### Maintenance of Fish Stocks and Embryo Collection

Breeding wild-type zebrafish (*Danio rerio*) (AB) were maintained and embryos raised under standard library conditions [19]. Embryos were collected and staged as described [20].

### Cloning of Zebrafish *Foxo3b*

Zebrafish *foxo3b* gene (GenBank accession numbers NM_131085) was amplified using the primers 5′-CACGCTCTAGAATGGCAGAGACAACCCT-3′ and 5′-ATATGGATCCTCAGCCTGGCACCCAACT-3′. Total RNA was isolated from zebrafish embryos using TRIzol reagent (Invitrogen), and cDNA was synthesized by using the RevertAid™ first strand cDNA synthesis Kit (Fermentas). The complete coding sequence was PCR amplified and subcloned into the pCGN-HAM vector (provided by William Tansey), and then sequence verified.

### Whole Mount In-situ Hybridization

The probes for identifying zebrafish *foxo3a, foxo3b, six3b, opl, cdx4, pax6* and *foxi1* were amplified from cDNA pools using the appropriate sets of primers (Table S1). The probes for *ved, vox, vent,* and *flh* were generous gifts from Dr Y. Sun (Institute of Hydrobiology, Chinese Academy of Sciences, Wuhan). The probes for *sqt*, *wnt8a, tbx5* and *bmp2b* were kindly provided by Dr Z. Yin (Institute of Hydrobiology, Chinese Academy of Sciences, Wuhan). Dr. T. Whitefield (Medical Research Council Centre for Developmental and Biomedical Genetics, Sheffield, UK) generously provided the probe for *nkx5.1*. The probe for *gata2* was kindly provided by Dr Tingxi Liu (Institute of Health Sciences, Shanghai). The probes for *gsc* and *bmp4* were described previously [18]. The procedure for whole-mount *in situ* hybridization was performed as described previously [21].

### Morpholino and mRNA Injection and Rescue Experiments

The morpholino antisense oligonucleotides (MOs) were obtained from Gene Tools: *foxo3b* -ATG-MO (5′-TGGCTCCAGGfGTTGTCTCTGCCATC-3′); *foxo3b*-SP-MO (5′- TGGAGATGCACTGCGCTTACCTTCC-3′); STD (standard)-MO (5′-CCTCTTACCTCAGTTACAATTTATA-3′); *β-catenin1*-MO (5′- CTGGGTAGCCATGATTTTCTCACAG -3′); *β-catenin2*-MO (5′- CCTTTAGCCTGAGCGACTTCCAAAC -3′). They were resuspended and injected as described previously [18].

A fragment of *foxo3b* containing 5′-UTR and N-terminus was cloned into pEGFP-N1 (Clontech) to generate wild-type *foxo3b* to validate the efficiency of *foxo3b-*MO. The primers were: 5′-ACATCTCGAGCACTGCCTATCTAACTTCGACC-3′ and 5′-ACATGGATCCCCATGCATTCCTCCTTGAAGAT-3′. The GFP-tagged mutated *foxo3b* was generated by PCR using a forward primer with 6 mismatched nucleotides: 5′-ACATCTCGAGATGGGTGAATCTACTCTGGAGCCACTGT-3′ (mismatched nucleotides are underlined). To further validate the specificity of *foxo3b-*MO and avoid quenching effect of *foxo3b* mRNA, we used a primer to introduce 5 mismatched nucleotides in the zebrafish *foxo3b* mRNA without changing the amino acid sequence, the primer was 5′-CACGAAGCTTATGGCTGAAACTACATTGGAGCCACTG-3′ (mismatched nucleotides are underlined). Dn*TCF* construct was kindly provided by Dr Y. Sun (Institute of Hydrobiology, Chinese Academy of Sciences, Wuhan). Dn*TCF* is a dominant negative form of *TCF3*, deleting the first 47 amino acids of β-catenin-binding domain. Capped mRNAs were synthesized using the AmptiCap SP6 High Yield message maker kit (Epicenter Biotechnologies). The synthetic mRNAs were diluted into different concentrations, and co-injected with morpholino to determine the optimal concentration that could rescue the defects of the morpholino-injected embryos effectively. capped mRNAs were injected into one-cell stage embryos at 1–2 ng. All of the microinjection was performed using a Harvard Apparatus PLI-100.

### Semi-quantitative RT-PCR

Total RNA was isolated from 30 whole embryos using TRIzol reagent (Invitrogen) at different developmental stages. Oligo-dT-primed cDNA was synthesized by using RevertAid™ first strand cDNA synthesis Kit (Fermentas) and random primers were used to reverse transcribe 2 µg RNA. Separate reactions were set up with primer pairs for *foxo3b* and *18s* RNA in the presence of SYBR green. All amplifications were performed using a two step temperature profile with annealing and extension at 60°C. Each sample was run in triplicate. Differences were calculated according to the ΔΔCt relative quantitation method (Applied biosystems) using the *18s* RNA as calibrator. The primers for the zebrafish *foxo3b* were 5′-CCAAGCACCTCTACATCTC-3′ and 5′-CTGTGAGAGACCAGCGAAT-3′. The primers for zebrafish *18s* were 5′-GAGAAACGGCTACCACATCC-3′ and 5′-CACCAGACTTGCCCTCCAA-3′.

### Validation of Splice Morpholino

Total RNA was isolated from 50 whole embryos using TRIzol reagent (Invitrogen) at bud stage. Oligo-dT-primed cDNA was synthesized by using RevertAid™ first strand cDNA synthesis Kit (Fermentas) and random primers were used to reverse transcribe 2 µg RNA. PCR was performed with the following primer sets: *Foxo3b*-p1-F, GTGAGTTACTGCTGGTGATGC; *Foxo3b*-p2-R, TCTTCAAGGAGGAATGCATG; *β-actin*-F, GATGATGAAATTGCCGCACTG; *β-actin-*R, ACCAACCATGACACCCTGATGT. *β-actin* was used as an internal control.

### Plasmid Construction

The vectors including pCGN-HAM (provided by William Tansey), pCMV-Flag2C (Stratagene), pEGFP-N1, pM-RFP and pM (Clontech) were used for cloning. Full-length cDNAs of zebrafish *foxo3b*, *β-catenin1* and *β-catenin2* (provided by Eric Weinberg) were subcloned into pCGN-HAM, pCMV-Flag2C, pEGFP-N1, pM-RFP or pM vectors to generate HA-*foxo3b,* HA-*β-catenin1,* HA-*β-catenin2,* Flag-*β-catenin1,* Flag*-β-catenin2,* pM-*β-catenin1,* pM*-β-catenin2,* GFP-*β-catenin1*, GFP-*β-catenin2*, RFP-*foxo3b*. All constructs were verified by sequencing.

### Luciferase Reporter Assays

Human embryonic kidney 293T cells were cultured in Dulbecco's modified Eagle's medium (DMEM) containing 10% fetal bovine serum (HyClone). Cells were seeded for 24h before transfection in 24-well plates, and were transfected with the mixture of plasmids (200 to 400 ng) by Lipofectamine 2000. pTK-*Renilla* luciferase reporter (10 ng) was used as an internal control. Similarly, the embryos were injected with the combined plasmids (250 pg per embryo) for luciferase assays. pFR-luc vector was purchased from Stratagene. The luciferase activity was determined at 24–30 hours post transfection or 11 hours after injection using the Dual-luciferase Reporter Assay System (Promega). The relative light units were measured using a luminometer (Sirius, Zylux Corporation, Oak Ridge, TN). Data were normalized by *Renilla* luciferase enzyme activity. Data are reported as mean ± SEM of three independent experiments performed in triplicate. The statistical analysis (paired t-test) was performed using GraphPad Prism 5.

### Fluorescence Microscopy

Human HeLa cells were transfected with different combinations of zebrafish RFP- *foxo3b*, GFP-*β-catenin1*, GFP-*β-catenin2* or empty RFP vectors. 24–30 hours after transfection, cells were directly observed under a Nikon T-2000 Eclipse inverted fluorescent microscope (Nikon Instruments, Melville, NY).

### Immunoprecipitation and Western Blot

Human embryonic kidney 293T cells and mouse L cells (with constitutively expressed *wnt3a*) were cultured in Dulbecco's modified Eagle's medium (DMEM). For immunoprecipitation assays, 293T cells were transfected with different combinations of HA-*foxo3b,* Flag-*β-catenin1,* Flag*-β-catenin2* or empty vectors (2–10 µg). 7 hours after transfection, the condition medium of mouse L cells was collected and added appropriately to 293T cells. After 24 hours, the 293T cells were washed with ice-cold PBS buffer and then lysed in RIPA (radioimmune precipitation) buffer containing 50 mM Tris, pH 7.4, 1% NP-40, 0.25% Na-deoxycholate, 1 mM EDTA, pH 8.0, 150 mM NaCl, 1 mM NaF, 1 mM PMSF (phenylmethylsulphonyl fluoride), 1 mM Na_ 3_VO_4_ (sodium orthovanadate) and 1∶100 dilution of protease inhibitor cocktail (Sigma). After incubation on ice for 1 h, lysates were centrifuged for 15 min at 10,000 g at 4°C, and supernatant was incubated with monoclonal anti-HA agarose conjugate beads (sigma) for 6 h or over-night at 4°C. The immunoprecipitates were washed 3 times with RIPA buffer. Immunoprecipitates (IP) and whole cell lysate (WCL) were boiled with 1x SDS sample buffer, separated on SDS-PAGE and transferred to PVDF membrane (Millipore). Western blot analysis was performed as described previously using the indicated antibodies [22].

## Results

### Expression of *Foxo3b* during Zebrafish Embryogenesis

**Figure 1:**
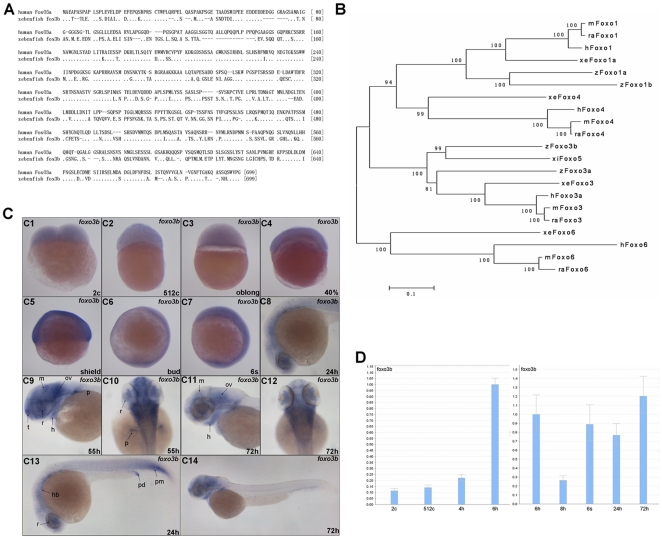
Sequence comparison of zebrafish *foxo3b* with other FOXOs and developmental expressing patterns of zebrafish *foxo3b*. (**A**) Sequence alignment of zebrafish foxo3b and human FOXO3a protein. (**B**) Neighbor-Joining Analysis of vertebrate FOXO protein sequences. Phylogenetic analysis was conducted using MEGA version 5 (Tamura, Peterson, Stecher, Nei, and Kumar 2011). h*Foxo3a* (*Homo sapiens*, Accession number NM_001455), h*Foxo6* (XM_002342102), h*Foxo4* (NM_005938), h*Foxo1* (NM_002015), m*Foxo3* (*Mus musculus*, NM_019740), m*Foxo6* (NM_194060), m*Foxo4* (NM_018789), mFoxo1 (NM_019739), ra*Foxo3* (*Rattus norvegicus*, NM_001106395), ra*Foxo6* (XM_001057233), ra*Foxo4* (NM_001106943), ra*Foxo1* (NM_001191846), z*Foxo3b* (*Danio rerio*, NM_131085), z*Foxo3a* (NM_001009988), z*Foxo1a* (NM_001077257), z*Foxo1b* (NM_001082857), xe*Foxo3* (*Xenopus laevis*, NM_001092949), xe*Foxo6* (NM_001159282), xe*Foxo4* (FJ811896), xe*Foxo1a* (NM_001092948), xi*foxo5* (*Xiphophorus maculates*, AY040320). NJ bootstrap values were shown on the branches. (**C**) The expression pattern of zebrafish *foxo3b* during embryogenesis. (C1–C3) The expression of *foxo3b* was detected at 2-cell stage embryos, and became weaker at oblong stage. (C4, C5) *Foxo3b* was ubiquitously expressed at 40% epiboly stage, a stronger expression was observed at shield stage. (C6, C7) *Foxo3b* became weaker at bud stage; at 6-somite stage, its expression level almost recovered to that of shield stage embryos. (C8, C13) *Foxo3b* was observed in the developing eye, hindbrain and posterior mesoderm by 24 hpf. (C9, C10) By 55 hpf, *foxo3b* expression was confined to the anterior central nervous system (CNS), with weak expression in the heart. (C11, C12, C14) By 72 hpf, *foxo3b* expression became weaker, but continued in the CNS and heart. C1–C4, lateral view; C5, lateral view with dorsal to the right; C6, C7, lateral view with anterior on top; C8, C9, C11, C13, C14, lateral views with anterior to the left; C10, C12, dorsal views with anterior on top; r, retina; p, pectoral fin bud; hb, hindbrain; pm, posterior mesoderm; pd, pronephric duct; h, heart; t, telencephalon; ov, otic vesicle; m, mesencephalon; c, cell; s, somite; h, hours post-fertilization (hpf). (**D**) Relative RNA expression levels as determined by semi-quantitative RT-PCR. For each stage, oligo dT-primed cDNA was used as template for three separate PCR amplifications using primers for *foxo3b* and *18s* (internal control), *foxo3b* reached a high expression level at shield stage. For quantitative purpose, mRNA expression levels were normalized to 6 hpf (1.00).

There are two orthologues of mammalian *FOXO3* gene in zebrafish, *foxo3a* and *foxo3b*. Zebrafish *foxo3b* was initially identified and named as zFKHR/*foxo5* [17]. Zebrafish *foxo3b* protein shared 55% identity to that of human *FOXO3a* (Fig. 1A). Neighbor joining phylogenetic analysis of vertebrate FOXO protein sequences showed that zebrafish *foxo3b* was part of a highly conserved branch including human, mouse, rat, Xenopus, and Xiphophorus *FOXO3* gene. Zebrafish *foxo3b* and Xiphophorus *foxo5* branched off somewhat earlier than zebrafish *foxo3a* (Fig. 1B).

Firstly, we employed *in situ* hybridization to check *foxo3b* expression pattern during embryogenesis and found that the *foxo3b* transcripts could be detected at 2-cell stage (Fig. 1C1). Its expression sustained until oblong stage, then a little bit decreased (Fig. 1C3). At 75% epiboly, another dramatic reduction of *foxo3b* expression was observed (data not show). Until 6-somites stage, the *foxo3b* transcripts were distributed ubiquitously among the whole embryo (Fig. 1C7). By 24 hpf, the expression of *foxo3b* was observed in brain (mainly in retina and hindbrain) and posterior mesoderm (Fig. 1C8 and C13). By 55 hpf, the strongest signals were predominantly detected in the central nervous system, including brain, retina, otic vesicle, and floor plate (Fig. 1C9 and C10), and became weaker by 72 hpf (Fig. 1C11, C12 and C14). Moreover, we employed semi-quantitative RT-PCR method to further determine relative levels of *foxo3b* transcripts at several key stages during embryogenesis. The results indicated that *foxo3b* transcripts could be detected at 2-cell stage (2c), but the expression level was relative low (column 1 from left to right in Fig. 1D). *Foxo3b* expression reached a higher level at 6 hpf, and dropped dramatically at 8 hpf. These results were consistent with that revealed by *in situ* hybridization. The detection of pre-MBT *foxo3b* expression implied that *foxo3b* might have important role in early embryogenesis.

### *Foxo3b* is Required for the Formation of Axis and Brain

**Figure 2:**
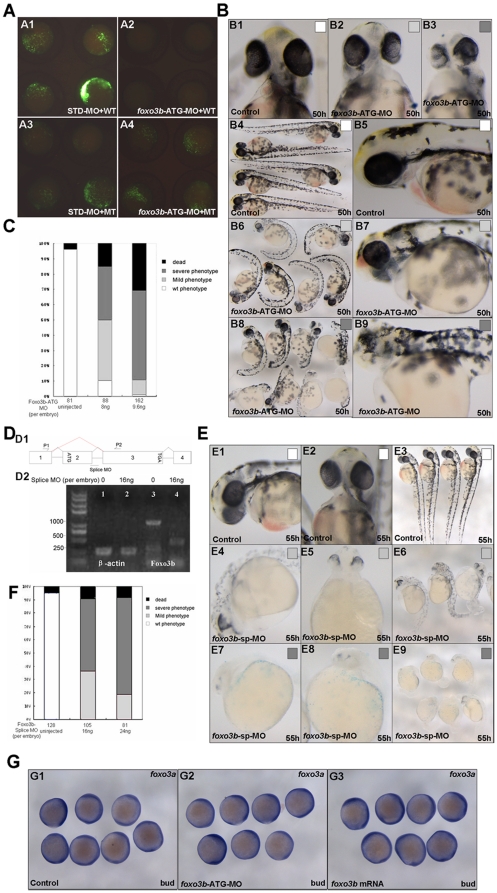
Knockdown of *foxo3b* results in defects in body axis and brain. (**A**) Validation of *foxo3b* ATG-blocking morpholino (*foxo3b*-ATG-MO). A1, embryos were injected with STD-MO (8 ng per embryo, control) and a wild-type *foxo3b*-GFP fusion protein expression vector (WT) and then examined by fluorescence microscopy; A2, embryos were injected with *foxo3b*-ATG-MO (8 ng per embryo) and a wild-type *foxo3b*-GFP fusion protein expression vector (WT); A3, embryos were injected with STD-MO and a mutated *foxo3b*-GFP fusion protein expression vector (MT); A4, embryos were injected with *foxo3b*-ATG-MO and a mutated *foxo3b*-GFP fusion protein expression vector (MT). A1-A4, bud stage. (**B, C**) Morphology of representative morphants in *foxo3b*-ATG-MO injected embryos. The morphants had shorter body length, abnormal brain and heart at 50 hpf. Black box, dead embryos at 24 hpf; B3, B8, B9, dark gray box, embryos with defects at 50 hpf characterized by severe phenotype: no blood circulation, severely reduced body length and thinner brain; B2, B6, B7, light gray box, embryos with mild phenotype; B1, B4, B5, white box, un-injected wild-type embryos. B1-B3, front views; B4, B6, B8, lateral views; B5, B7, B9, lateral views with anterior to the left. (**D**) Validation of *foxo3b* splice-blocking morpholino (*foxo3b*-SP-MO). D1, *Foxo3b* exon/intron structure. *Foxo3b*-SP-MO can alter splicing of *foxo3b* mRNA, which results in the production of an aberrantly spliced message (as showed by red line). D2, The injection of *foxo3b*-SP-MO results in the production of a truncated mRNA (440 bp). The embryos were collected at bud stage, and *β-actin* was used as an internal control. (**E, F**) Morphology of splice-MO injected embryos. By 55 hpf, the morphants showed defects similar to that of *foxo3b*-ATG-MO injected embryos. (**G**) The expression level of *foxo3a* was not altered in *foxo3b*-knockdown or *foxo3b* over-expressed embryos. Embryos were injected with 8 ng *foxo3b*-ATG-MO (G2) or 1 ng *foxo3b* mRNA (G3) at 1-cell stage. Wild-type embryos were used as control. G1-G3, bud stage, lateral views.

To determine the roles of *foxo3b* during zebrafish embryogenesis, we knocked down its expression using a morpholino targeting the translation initiation region of zebrafish *foxo3b* (*foxo3b*-ATG-MO). Firstly, we evaluated the efficiency of *foxo3b*-ATG-MO by injecting 1-cell stage zebrafish embryos with either STD-MO (standard morpholino) or *foxo3b-*ATG-MO, together with a vector expressing the truncated target protein tagged with GFP at the carboxyl terminus (WT) (Fig. 2A1 and A2). We also injected the indicated morpholinos with a vector expressing a mutated form of GFP-tagged truncated *foxo3b* (MT) (Fig. 2A3 and A4), which had 6 mismatched nucleotides in the *foxo3b*-ATG-MO targeted sequence. The results showed that *foxo3b* morpholino (8 ng per embryo) successfully blocked expression of *foxo3b*-GFP (WT) (Fig. 2A2), but not the expression of the mutated *foxo3b*-GFP (MT) (Fig. 2A4).

The embryos injected with *foxo3b*-ATG-MO showed the phenotypes characterized with shortened body axis and thinner brain by 50 hpf (hours post fertilization) (Fig. 2B). The morphants could be classified into two classes: class I (moderate defect) showing shortened body axis, smaller eyes and thinner head (Fig. 2B2, B6 and B7); class II (serious defect) showing serious abnormality of brain and eyes, some even without forebrain and eyes (Fig. 2B3, B8 and B9). To determine if the changes in the morphology occurred in a dose-dependent manner, we injected 1-cell stage embryos with varying concentrations of *foxo3b*-ATG-MO (8 ng and 9.6 ng per embryo respectively). We then evaluated the embryos for viability by 24 hpf and for defects by 50 hpf. The results showed that *foxo3b*-ATG-MO had a dose-dependent effect (Fig. 2C).

To further confirm the specificity of phenotypes exhibited in *foxo3b*-ATG-MO morphants, we targeted the *foxo3b* gene with splice-blocking MO (*foxo3b*-SP-MO), and tested whether it could cause phenotypes similar to that exhibited in *foxo3b*-ATG-MO morphants. *Foxo3b* exon/intron structure was showed in Figure 2D1. *Foxo3b*-SP-MO was designed based on 25 base pair sequence complementary to the linkage site of exon 2 and exon 3 in *foxo3b*. Firstly, we tested whether the splice-blocking MO could indeed alter splicing of *foxo3b* mRNA as expected. By RT-PCR, we found that injection of *foxo3b*-SP-MO into 1-cell stage embryos resulted in production of an aberrantly spliced message with the size matched to the prediction (440 bp) (Fig. 2D1 & D2). The phenotypes exhibited in *foxo3b*-SP-MO morphants could also be classified into two classes as that of the *foxo3b*-ATG-MO morphants (Fig. 2E4-E9). In addition, *foxo3b*-SP-MO had a dose-dependent effect and produced more severe defects with higher concentration (16 ng and 24 ng per embryo respectively) (Fig. 2F). Thus, the phenotypes exhibited in *foxo3b*-SP-MO morphants phenocopied to that of *foxo3b*-ATG-MO morphants. Those observations suggested that both *foxo3b*-ATG-MO and *foxo3b*-SP-MO could specifically knockdown endogenous *foxo3b* expression efficiently.

In addition, *foxo3b*-ATG-MO morphants and *foxo3b*-SP-MO morphants displayed similar phenotypes implied that both maternal and zygotic *foxo3b* functioned importantly during embryogenesis. However, two orthologues of mammalian *FOXO3*, *foxo3a* and *foxo3b*, existed in zebrafish genome, we wonder whether the phenotype of *foxo3b* morphants resulted from the expression change of *foxo3a* in embryos, so we detected the expression of *foxo3a* after either *foxo3b* knockdown or over-expression. As shown in Figure 2G, the expression level of *foxo3a* was not altered in either *foxo3b*-knockdown or over-expressed embryos. These results further suggested that the phenotype of *foxo3b* morphants was specifically caused by *foxo3b* knockdown, which was further refined by the following rescue experiments.

### *Foxo3b* is Required for Neuroectoderm Formation and Neural Tube Patterning

**Figure 3:**
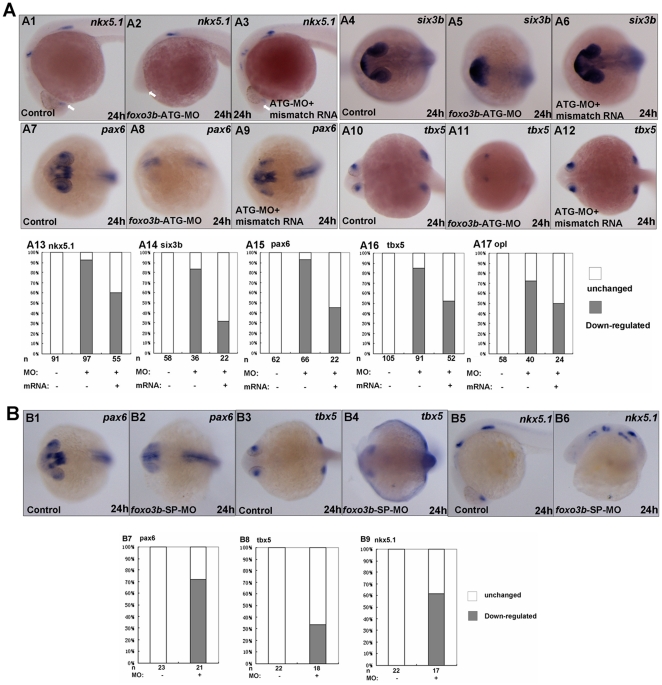
Loss of *foxo3b* function results in anterior defects. (**A**) The *foxo3b*-ATG-MO injected embryos showed remarkable loss of expression of anterior neural markers by 24 hpf, which could partially be rescued by co-injection of *foxo3b* mismatch mRNA. (A1–A3, A13) The expression of *nkx5.1* at the telencephalon (indicated by white arrows) was dramatically reduced in *foxo3b*-ATG-MO injected embryos. Co-injection of *foxo3b* mismatch mRNA could partially restore its expression at the telencephalon. (A4-A6, A14) *Six3b* was specifically expressed at the telencephalon and eyes. Loss of *foxo3b* function resulted in abnormal expression pattern of *six3b*, which was restored by co-injection of *foxo3b* mismatch mRNA. (A7–A9, A15) *Pax6* expression at the forebrain and eyes decreased greatly in *foxo3b* morphants. Co-injection of *foxo3b* mismatch mRNA partially restored its expression. (A10–A12, A16) Expression of *tbx5* at the retina was dramatically reduced in *foxo3b*-knockdown embryos. Its expression was rescued by co-injection of *foxo3b* mismatch mRNA. (A17) *Opl* expression at the telencephalon was reduced in *foxo3b* morphants compared to control embryos, which was efficiently rescued by co-injection of *foxo3b* mismatch mRNA. Embryos were injected with 8 ng *foxo3b*-ATG-MO or 125 pg *foxo3b* mismatch mRNA,wild-type embryos were used as control. A1-A3, lateral views with anterior to the left; A4-A12, dorsal views with anterior to the left; A1-A17, 24 hpf. (**B**) The *foxo3b-*SP-MO injected embryos exhibited anterior defects similar to that of *foxo3b*-ATG-MO injected embryos. (B1–B2, B7) The expression of *pax6* at the telencephalon and eyes was reduced in *foxo3b*-SP-MO injected embryos. (B3–B4, B8) *Tbx5* expression at the eyes decreased in *foxo3b*-knockdown embryos. (B5–B6, B9) Loss of zygotic *foxo3b* function resulted in reduction of *nkx5.1* expression at the telencephalon. Embryos were injected with 16 ng STD-MO (control) or 16 ng *foxo3b*-splice-MO. B1–B4, dorsal views with anterior to the left; B5-B6, lateral views with anterior to the left; B1-B9, 24 hpf.

In *foxo3b* morphants, the embryos displayed abnormality in head formation, such as smaller forebrain and eyes. This fact prompted us to examine whether *foxo3b* knockdown would cause defects in neural tube formation. We examined several markers to test whether *foxo3b* had an essential role in neural induction. *Nkx5.1* (also known as homeobox 3 gene), is expressed in the central nervous system. A remarkable loss of *nkx5.1* expression at the telencephalon was observed in *foxo3b* morphants (indicated by white arrows, Fig. 3A2). Another forebrain neural keel marker *six3b* (sine oculis homeobox homolog 3b), specifically expressed at the telencephalon and eyes, exhibited an abnormal expression pattern in *foxo3b* morphants (Fig. 3A5). Moreover, neuroectoderm marker *pax6* (paired box gene 6a), expressed dominantly in forebrain and eyes, was reduced dramatically in *foxo3b* knockdown embryos (Fig. 3A8). *Tbx5* (T box gene 5) is expressed in the eyes, heart and pectoral fins during embryogenesis [23]. As revealed by *tbx5* staining, some *foxo3b* morphants failed to develop one or two eyes, some developed smaller eyes in *foxo3b* morphants (Fig. 3A11). In addition, the *foxo3b* morphants seemed to have much thinner head (Fig. 3A).

Meanwhile, we conducted rescue experiments to validate the efficiency and specificity of *foxo3b*-MO. To avoid quenching, we used *foxo3b* mismatch mRNA in which the N-terminus of *foxo3b* mRNA was altered so that it was no longer complementary to the sequence of *foxo3b*-ATG-MO. As shown in Figure 3A, co-injection of *foxo3b* mismatch mRNA efficiently restored normal expression of all neuroectoderm markers (*nkx5.1, six3b, pax6, tbx5* and *opl*) at the forebrain and eyes.

As expected, injection of *foxo3b*-SP-MO resulted in anterior defects similar to that of *foxo3b*-ATG-MO morphants. Several neuroectoderm markers, *tbx5, nkx5.1* and *pax6*, showed obviously reduction of expression at forebrain and eyes in *foxo3b*-SP-MO morphants (Fig. 3B). Taken together, these data suggested that *foxo3b* was required for anterior neuroectoderm formation and neural tube patterning.

However, in the posterior region, particularly in *foxo3b* splice morphants, the expression of *tbx5* increased, the expression of *pax6* in posterior neuroectoderm including hindbrain and spinal chord also increased obviously, but the expression of *nkx5.1* in posterior neuroectoderm only displayed disorganized, which was not reduced as that in telencephalon (Fig. 3B). As reported, zygotic Wnt8/β-catenin is required for posteriorization of the neuroectoderm and the formation of the posterior mesoderm [24], [25], [26]. These observations indicated that *foxo3b* might negatively regulate zygotic Wnt8/β-catenin signaling.

### Loss of *Foxo3b* Leads to Defects in Dorsal-ventral Patterning during Early Embryogenesis and *Foxo3b* Affects Wnt/β-catenin Signaling

The majority of *foxo3b* morphants displayed a disorganization of the head and shortened body axis, thus, we selected several marker genes involved in DV patterning to further reveal the role of *foxo3b* in early zebrafish axis formation in this study.

**Figure 4:**
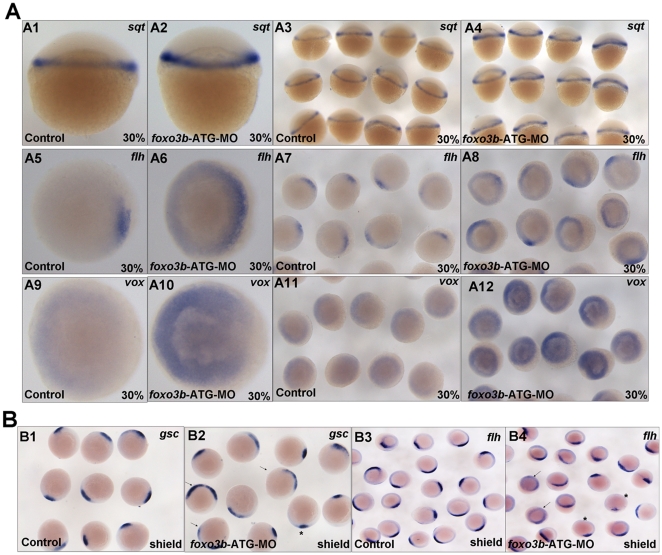
Knockdown of *foxo3b* leads to defects in DV patterning during early embryogenesis. (**A**) A1–A4, *sqt* expression increased in *foxo3b*-MO injected embryos compared to control embryos. A5–A8, presumptive organizer marker *flh* expanded at *foxo3b*-knockdown embryos. A9–A10, *foxo3b* knockdown caused ectopic *vox* expression at 30% epiboly. (**B**) The expression of presumptive organizer marker *gsc* and *flh* in *foxo3b*-MO injected embryos. The arrows identified embryos with expanded expression (B2, 74%, n = 23; B4, 52%,n = 21, respectively) and asterisks identified embryos with decreased expression. Embryos were injected with 8 ng STD-MO (control) or 8 ng *foxo3b*-ATG-MO. A1–A4, lateral views; A5–A12, B1–B4, animal pole views; A1–A12, 30% epiboly; B1–B4, shield stage.

At 30% epiboly, the knockdown of *foxo3b* caused elevated expression of *sqt* (squint) (Fig. 4A1-A4), a nodal-related ligand, which is the main direct target of maternal Wnt/β-catenin signaling [27], [28]. *Foxo3b* morphants also showed expanded expression of organizer marker gene *flh* (floating head) (Fig. 4A5–A8). These results indicated that knockdown of *foxo3b* in embryos increased maternal β-catenin signaling activity and promoted organizer formation. In addition, a significant increase of *vox* expression was also observed in *foxo3b* morphants at 30% epiboly (Fig. 4A9–A12). As a member of the ventrally expressed homeobox genes, *vox* gene is a direct target gene of *β-catenin* [7] and first expressed ubiquitously after the MBT by maternal factors [29]. High level of *vox* expression is maintained by Wnt8/β-catenin signaling at early gastrulation stage. At later gastrulation, its expression is regulated by BMP signaling. These observations implied that *foxo3b* knockdown could affect zebrafish dorsal-ventral patterning during early embryogenesis probably through antagonizing Wnt**/**β-catenin signaling.

Wnt/β-catenin signaling is essential for organizer formation and DV patterning [4], [7], [30]. In addition, Wnt/β-catenin signaling can affect discrete domains of gene expression along the anterior-posterior (AP) axis of the neural plate and plays a role in establishing neural tube compartments along the axis [12]. In this study, the fact that the loss of *foxo3b* could result not only in defects of neuroectoderm formation, neural tube patterning and dorsal-ventral patterning, but also in mis-expression of Wnt target genes, strongly suggested that *foxo3b* could affect Wnt**/**β-catenin signaling.

As reported, *wnt8* directly regulates the transcription of *vent* and *vox*, starting at the blastula/gastrula transition (30/40% epiboly). The maintenance of high levels of *vent* or *vox* expression by *wnt8* is required for the repression of organizer genes on the ventral side of the embryo [7]. At gastrula stage, β-catenin activity influences in both dorsal and ventro-lateral discrete domains, to mediate Wnt ligand signaling [4]. Thus, the organizer genes expression are affected by both the positive influence of maternal and the negative influence of ventro-lateral zygotic Wnt/β-catenin signaling at 50% epiboly [31]. Consistent with this notion, the *foxo3b* morphants at shield stage displayed mixed expression of organizer genes *flh* and *gsc* (goosecoid): 52% and 74% of morphants showed expanded *flh* and *gsc* expression respectively (arrow indicated, Fig. 4B2 and B4), while 10% and 9% of morphants showed reduced *flh* and *gsc* expression (asterisk indicated, Fig. 4B2 and B4). However, we could not rule out a possibility that the mixed expression of *flh* and *gsc* in shield stage morphants might also resulted from the incomplete penetration of morpholinos or delayed embryogenesis process, which is needed to be further defined.

As showed above, *foxo3b* was maternally expressed and maternal β-catenin direct target gene *sqt* displayed enhanced expression. Moreover, other canonical Wnt/β-catenin signaling markers also exhibited mixed expression pattern at shield stage. Taken together, these observations implied that zebrafish *foxo3b* might serve as a main partner participating in negatively regulating both maternal and zygotic Wnt/β-catenin signaling.

### *Foxo3b* Antagonizes Wnt/β-catenin Signaling during Dorsal-ventral Patterning

**Figure 5:**
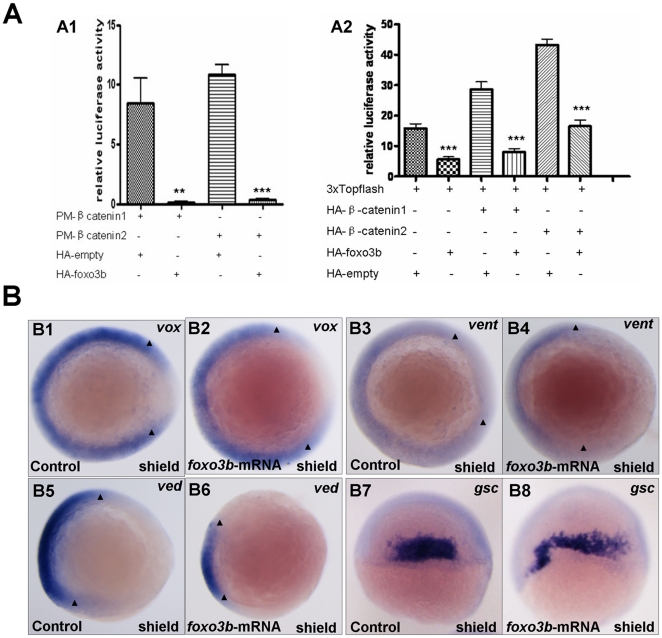
*Foxo3b* gain-of-function inhibits Wnt/β-catenin signaling in embryos. (**A**) *Foxo3b* inhibited β-catenin/T cell factor activity in embryos. A1, 1-cell stage embryos were injected with a mixture of plasmids as indicated, together with pFR-luc as a reporter gene and pTK-*renilla* as an internal control; luciferase activity was measured after 11h. Date presented are the average (±SEM) of four independent experiments. A2, 1-cell stage embryos were injected with 3xTOPFlash, and the plasmids as indicated, together with pTK-*renilla* as an internal control; luciferase activity was measured after 11 h, performed in triplicate. “**” indicates p<0.01; “***” indicates p<0.001. (**B**) Gain-of-function of *foxo3b* resulted in suppression of Wnt/β-catenin signaling in embryos. B1-B2, *foxo3b* over-expressed embryos showed reduced *vox* expression (arrowheads in B1 and B2) compared to wild-type. B3-B6, the expression of ventral marker *vent* and *ved* (domain width indicated by arrowheads) decreased in 70% (n = 20) and 75% (n = 16) of *foxo3b*-ATG-MO-injected embryos respectively. B7-B10, the expression domain of dorsal marker *gsc* expanded in most *foxo3b* over-expressed embryos. Embryos were injected with 2 ng GFP mRNA (control) or 2 ng *foxo3b* mRNA. B1-B6, animal pole views with dorsal to the right; B7-B8, dorsal views with anterior on top; B1-B8, shield stage.

To further verify that *foxo3b* could inhibit Wnt/β-catenin signaling, we checked whether *foxo3b* suppressed the transcriptional activity of zebrafish β-catenin. Firstly, we constructed an artificial transcription factor by fusing zebrafish full-length *β-catenin1/2* with Gal4 DBD (the corresponding expression plasmids were designated as pM*-β-catenin1* and pM*-β-catenin2*, respectively). Subsequently, we injected 1-cell stage embryos with pM*-β-catenin1/2,* HA*-empty* or HA*-Foxo3b*, together with pFR-luc (a Gal4-dependent promoter linked to the luciferase gene) as a reporter, and pTK-*Rellina* as an internal control. The luciferase activity was measured 11 hours after injection. The results indicated that *foxo3b* dramatically inhibited β-catenin transcriptional activity (p = 0.0069 for pM*-β*-*catenin1*, p<0.0001 for pM*-β*-*catenin2* in Fig. 5A1).

*β-catenin* induces transcription of Wnt target genes through binding to lymphoid enhancer factor/T cell factor in the nucleus. To test the inhibition of *foxo3b* on *β-catenin* transcriptional activity, we analyzed the effect of *foxo3b* on TCF-dependent transcription using 3xTOPFlash reporter (a β-catenin-dependent promoter which contains 3 copies of an optimal TCF-binding site) assays. As shown in Figure 5A2, TCF-dependent transcription was activated by endogenous zebrafish *β-catenin1/2*, and this activation was dramatically suppressed by over-expression of *foxo3b* (column 1 and 2 from left to right in Fig. 5A2); Over-expression of zebrafish *β-catenin1* or *β-catenin2* enhanced TCF-dependant transcription, which could also be suppressed by over-expression of *foxo3b* (column 3-6 from left to right in Fig. 5A2).

To further determine whether *foxo3b* could indeed antagonize Wnt/β-catenin signaling *in vivo*, we injected synthetic zebrafish *foxo3b* mRNA into 1-cell stage embryos, then assayed for Wnt target genes at shield stage. We observed the morphogenesis of embryos with ectopic *foxo3b* expression firstly, most embryos with ectopic *foxo3b* expression exhibited expansion of anterior brain, and curved body, partly with cyclopic eye of normal size (Data not shown), which is not totally opposite to the phenotype of *foxo3b* morphants. FOXO transcription factors are important mediator of the PI3K/Akt pathway and involved in a series of cellular functions [32]. As a transcriptional factor, FOXO can regulate multiple target gene expression, such as dLnR, d4EBP and Bim, which participate in modulating cell apoptosis and cell cycle [33]. In addition, FOXO can bind with other transcription factors, such as C/EBP beta, to affect their function [34]. Therefore, we assumed that ectopic expression of *foxo3b* might influence multiple signaling pathways in addition to Wnt/β-catenin signaling during early embryogenesis, resulting in complex phenotypes exhibited in *foxo3b* over-expressed embryos.

Over-expression of *foxo3b* resulted in reduction of ventral gene expression in most embryos. As shown in Figure 5, in 70% injected embryos (n = 20), *vent* expression domain was reduced obviously (Fig. 5B3 and B4), and 75% injected embryos (n = 16) displayed reduced *ved* expression arc (Fig. 5B5 and B6). Similarly, high frequency of *foxo3b* mRNA injected embryos showed reduced *vox* expression (Fig. 5B1 and B2). On the contrary, the dorsal marker gene *gsc*, displayed expanded expression pattern in most *foxo3b* over-expressed embryos (Fig. 5B7 and B8).

### *Foxo3b* is Required for Posterior Neuroectoderm and Mesoderm

**Figure 6:**
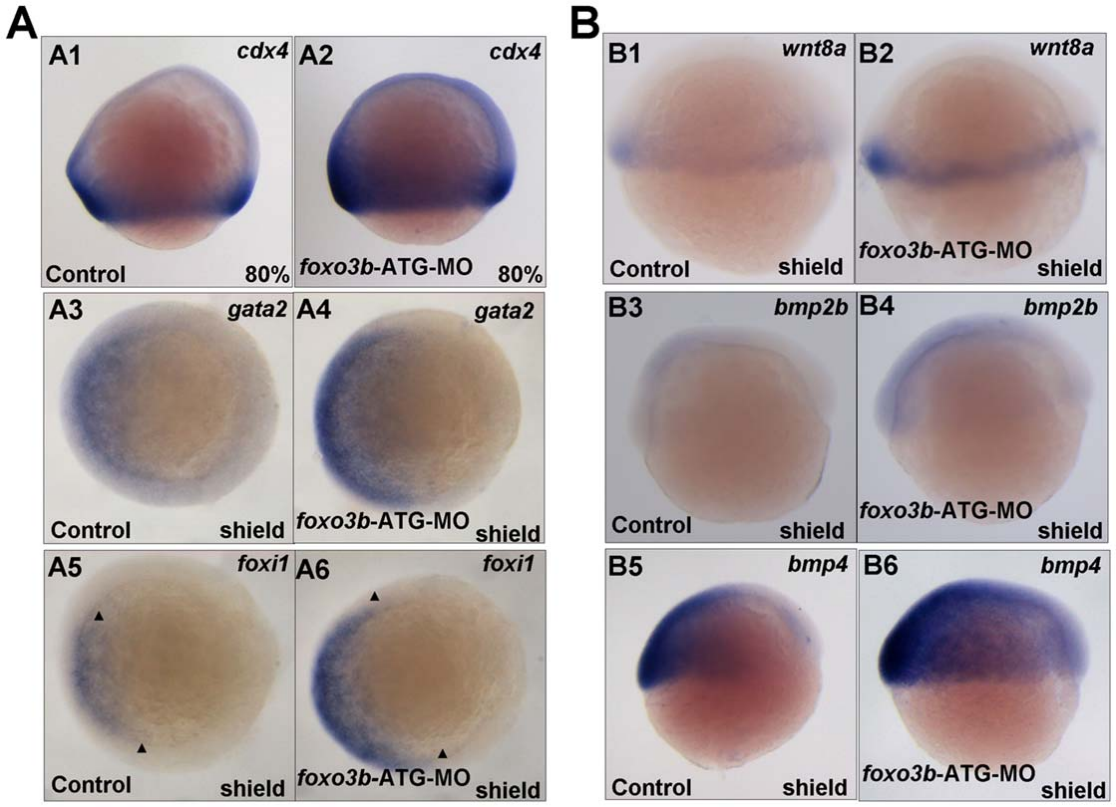
*Foxo3b* functions in posterior neuroectoderm formation and involves with BMP signaling in DV patterning. (**A**) *Foxo3b* affected posterior neuroectoderm formation and mesoderm induction. A1-A2, at 80% epiboly, *cdx4* expression expanded in *foxo3b*-MO injected embryos compared to control embryos. A3-A4, the expression of non-neural ectodermal marker *gata2* was up-regulated in *foxo3b*-knockdown embryos. A5-A6, ectoderm marker *foxi1* expanded in *foxo3b* morphants. (**B**) The expression of *wnt8* and *bmp* ligands in *foxo3b* morphants. B1–B2, *wnt8* expression increased in *foxo3b* morphants at shield stage. B3–B6, the expression of ventral markers *bmp2b/bmp4* was up-regulated in *foxo3b*-knockdown embryos at shield stage. Embryos were injected with 8 ng STD-MO (control) or 8 ng *foxo3b*-ATG-MO. A1–A2, lateral views with dorsal to the right; A3–A6, animal pole views with dorsal to the right; B1–B6, lateral views with dorsal to the right; A1–A2, 80% epiboly; A3–A6, B1–B6, shield stage.

Previous studies revealed that Wnt/β-catenin signaling is required for neural posteriorization by directly regulating *cdx4* to promote posterior *hox* gene expression [35]. As *foxo3b* morphants displayed increased Wnt/β-catenin signaling, we next examined whether posterior body formation was affected by *foxo3b* knockdown. Firstly, we checked expression of Wnt target gene *cdx4* (caudal type homeobox transcription factor 4/kugelig gene), which was required for posterior body formation. The result showed that almost all *foxo3b* morphants exhibited expanded expression of *cdx4* (Fig. 6A2). In addition, non-neural ectoderm marker *gata2* (GATA-binding protein 2a) and pan-ectoderm marker *foxi1* (forkhead box I1) expression were up-regulated in *foxo3b* morphants (Fig. 6A4 and A6), suggesting that *foxo3b* might also play important roles in regulating cell fate differentiation.

The above observations have showed that knockdown of *foxo3b* led to elevated Wnt/β-catenin signaling and resulted in defects in DV (dorsal-ventral) patterning and AP neural tube patterning. DV patterning of vertebrate embryos requires the concerted actions of the BMP and Wnt signaling, and Wnt signals cooperate with Bmp to regulate the formation of the posterior mesoderm [26]. To better understand the role of *foxo3b* and the underlying mechanism, we checked the expression of *wnt8*, *bmp2b* and *bmp4* (bone morphogenetic protein 2b and 4) in *foxo3b* knockdown embryos. Both *wnt8* and *bmp2b/bmp4* expression were up-regulated in *foxo3b* morphants (Fig. 6B). In addition, the up-regulation of *bmp2b* expression in *foxo3b* morphants could be rescued by co-injection of *foxo3b* mismatched mRNA (Fig. S1).

In addition, *wnt8* was also used as a ventro-lateral and posterior mesoderm marker during zebrafish embryogenesis to monitor the mesoderm induction in embryos [36], [37], [38]. The increased expression of *wnt8* in *foxo3b* morphants was consistent with the observation that *cdx4* exhibited expanded expression in posterior position. Wnt8/β-catenin signaling was reported to promote the formation of the ventro-lateral and posterior mesoderm and neuroectoderm [24], [25], so it was possible that enhanced Wnt/β-catenin signaling in *foxo3b* morphants resulted in increased ventro-lateral mesoderm marker *wnt8* expression. Furthermore, the increased *wnt8* expression might partially contribute to the anterior defects in *foxo3b* morphants by activating the canonical Wnt signaling.

### *Foxo3b* Mediated Anterior Neuroectoderm Formation and DV Patterning Through Maternal and Zygotic Wnt/β-catenin Signaling

**Figure 7:**
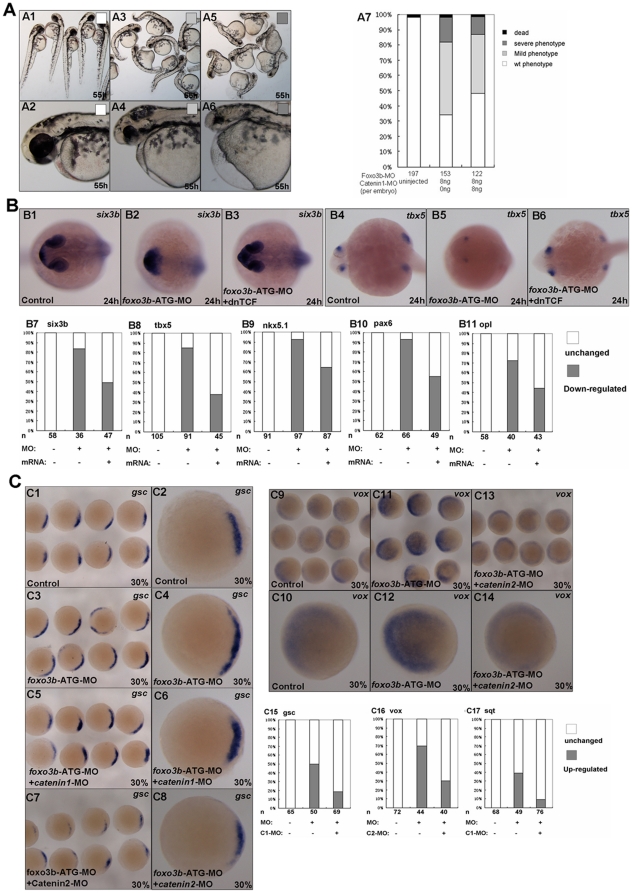
*Foxo3b* inhibits both maternal and zygotic Wnt/β-catenin signaling. (**A**) *Foxo3b* morphants could be rescued by knockdown of *β-catenin1*. Black box, dead embryos at 24 hpf; A5-A6, dark gray box, embryos with defects at 55 hpf characterized by severe phenotype (as described in Fig. 2B); A3-A4, light gray box, embryos with mild phenotype; A1–A2, white box, un-injected wild-type embryos. A1, A3, A5, lateral views; A2, A4, A6, lateral views with anterior to the left; A1–A7, 55 hpf. (**B**) Anterior defects caused by loss of *foxo3b* function could be rescued by co-injection of dn*TCF* mRNA. (B1–B3, B7) *Six3b* was expressed abnormally in *foxo3b* morphants, which could be rescued by co-injection of dn*TCF* mRNA by 24 hpf. (B4–B6, B8) *Tbx5* expression at the eyes was greatly reduced in *foxo3b*-knockdown embryos. Co-injection of dn*TCF* mRNA could efficiently restore its expression at the eyes. (B9–B11) *Nkx5.1, pax6* and *opl* expression at the anterior neural plate could also be efficiently rescued by co-injection of dn*TCF* mRNA. Embryos were injected with 8 ng *foxo3b*-ATG-MO or 10 pg dn*TCF* mRNA,wild-type embryos were used as control. B1–B6, dorsal views with anterior to the left; B1–B11, 24 hpf. (**C**) *Foxo3b* knockdown resulted in abnormal expression of early Wnt target genes, which could be rescued by co-injection of *β-catenin1/2* morpholino. (C1–C8, C15) The morphants showed increased expression of *gsc*, which could also be rescued by co-injection of *β-catenin1* MO. Co-injection of *β-catenin2* MO resulted in dramatically reduction of *gsc* expression (75%, n = 32) compared to control embryos. (C9-C14, C16) The expression level of *vox* increased dramatically in *foxo3b*-knockdown embryos, which could be partially rescued by co-injection of *β-catenin2* MO. (C17) At 30% epiboly, *sqt* expression was up-regulated in *foxo3b* morphants. Co-injection of *β-catenin1* morpholino efficiently reduced its expression. Embryos were injected with 8 ng *foxo3b*-ATG-MO or 8 ng *β-catenin1/2* morpholino,wild-type embryos were used as control. C1-C14, dorsal views; C1-C17, 30% epiboly.

Based on our above data, we proposed that defects of *foxo3b* morphants in DV patterning and neuroectoderm formation, might result from up-regulation of Wnt/β-catenin signaling. To verify our hypothesis, we performed rescue experiments by co-injection of *β-catenin1-*MO, *β-catenin2-*MO or dn*TCF* mRNA with *foxo3b*-ATG-MO. After injection, we scored embryos for expression of the neuroectoderm markers *six3b, tbx5*, *nkx5.1, pax6* and *opl* using *in situ* hybridization (Fig. 7B) as well as general morphological characteristics (Fig. 7A). Translation-blocking morpholinos targeting zebrafish *β-catenin1* and *β-catenin2* are designed as previously described [4]. We found that *β-catenin1* morpholino could partially rescue the anterior defects resulting from *foxo3b* knockdown (Fig. 7A), and *β-catenin2* morpholino could also rescue the phenotype of *foxo3b* morphants (data not show).

In addition, we tested whether dn*TCF* could rescue anterior defects in *foxo3b* morphants. As a dominant negative form of zebrafish *TCF3* (also known as *headless*), dn*TCF* is an effective suppressor of Wnt/β-catenin signaling. As expected, the reduction of neuroectoderm markers expression at the forebrain and eyes could be rescued by co-injection of dn*TCF* mRNA by 24 hpf. As shown in Figure 7B, 83.3% of *foxo3b* morphants displayed abnormal expression of *six3b*, this proportion dropped down to 49% after co-injection with dn*TCF* mRNA. In addition, 84.6% of *tbx5* expression at the eyes was greatly reduced in *foxo3b* morphants, after co-injection with dn*TCF* mRNA, only 37.8% of embryos displayed the reduced expression of *tbx5* at the eyes. Co-injection of dn*TCF* mRNA could also rescue the expression of *nkx5.1, pax6* and *opl* at the anterior neuroectoderm in *foxo3b* morphants. These results suggested that the defects of anterior neural domains in *foxo3b* morphants indeed resulted from up-regulation of Wnt/β-catenin signaling. Besides the anterior defects of *foxo3b* morphants could be rescued by co-injection of *β-catenin1*-MO *or β-catenin2*-MO (Fig. 7A), further proving that the role of *foxo3b* in affecting anterior neuroectoderm formation was mediated by wnt/β-catenin signaling.

At late blastula period, loss of *foxo3b* function in embryos resulted in up-regulation of several Wnt targets (*sqt, gsc*, and *vox*) (Fig. 7C), which might result from the elevation of maternal wnt signaling. Co-injection of *β-catenin1* morpholino successfully reduced dorsal markers genes *gsc* and *sqt* to normal level in *foxo3b* morphants at blastula stage. 38% of *foxo3b* morphants exhibited up-regulation of *gsc* expression, when co-injected with *β-catenin1* morpholino, the rate of *gsc* up-regulation was reduced to 18.8% (Fig. 7C6 and C15). Co-injection of *foxo3b*-ATG-MO with *β-catenin2* morpholino resulted in obviously reduced expression of *gsc* at 30% epiboly (Fig. 7C8). 38.8% of *foxo3b* morphants with up-regulation of *sqt* was reduced to 9.2% when co-injected with *β-catenin1* morpholino (Fig. 7C17). In addition, co-injection of *β-catenin2* morpholino successfully restored ventral marker *vox* expression to normal level at blastula stage. 69.7% of *foxo3b* morphants (n = 44) showed up-regulation of *vox* expression, which was dropped down to 30% after co-injection of *β-catenin2* morpholino (Fig. 7C14 and C16).

These results that the abnormal expression of marker genes involved in DV patterning or anterior neuroectoderm defects, resulting from *foxo3b* knockdown, could be successfully rescued by knockdown of *β-catenin1* or *β-catenin 2* suggested that *foxo3b* could inhibit both maternal and zygotic Wnt/β-catenin signaling.

In the rescue experiments, we could partially rescue the *foxo3b* morphants by injecting with dn*TCF* mRNA, *β-catenin1*-MO or *β-catenin 2-*MO, which further indicated that *foxo3b* could indeed affect embryogenesis by inhibiting both maternal and zygotic Wnt/β-catenin signaling. FOXO transcription factors act as downstream effectors of many important pathways, such as PI3K/Akt pathway, TGF-β pathway [32], [39], they can mediate multiple biological function by regulating different target genes [33], [34], [40]. Our rescue experiments also implied that *foxo3b* might affect other signaling pathways in addition to Wnt signaling during zebrafish embryogenesis.

### *Foxo3b* Interacts with *β-catenin 1* and *β-catenin 2*

**Figure 8:**
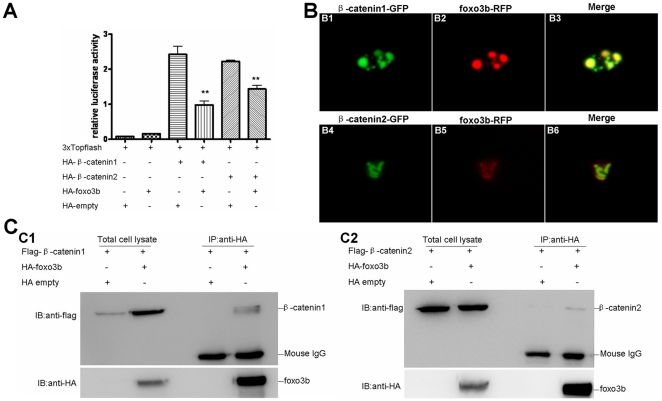
*Foxo3b* interacts with *β-catenin1/2* in 293T cell. (**A**) *Foxo3b* inhibited β-catenin/T cell factor activity in 293T cell line. 293T cells were transfected with 3xTOPFlash, and the plasmids as indicated, together with pTK*-renilla* as an internal control; luciferase activity was measured after 24h. Date presented were the average (±SEM) of three independent experiments, performed in triplicate. “**” indicates p<0.01. (**B**) *Foxo3b* co-localized with *β-catenin1/2*. HeLa cells were transfected with GFP-*β-catenin1* (B1-B3) or GFP-*β-catenin2* (B4–B6), together with RFP-*foxo3b*. Transfected cells were then obser*ved* using fluorescent microscopy after 24h. (**C**) *Foxo3b* interacted with *β-catenin1/2*. 293T cells were transfected with the indicated expressing plasmids. HA-*foxo3b* was immunoprecipitated, and binding of Flag-*β-catenin1* (C1) or Flag-*β-catenin2* (C2) was analyzed by immunoblotting.

Because *foxo3b* could inhibit the transactivity of artificial transcription factors, pM-*β-catenin1* and pM-*β-catenin2*, and previous studies had showed that mammalian FOXO competed with TCF to interact with β-catenin [16], these evidences prompted us to check whether *foxo3b* could interact with *β-catenin1* and *β-catenin*2. Firstly, we analyzed the effect of *foxo3b* on TCF-dependent transcription activity using 3xTOPFlash reporter in 293T cell. As showed in Figure 8A, over-expression of *foxo3b* could suppress β-catenin/TCF signaling significantly in 293T cell (p = 0.0049 for *β-catenin1*, p = 0.0019 for *β-catenin2* in Fig. 8A).

Subsequently, we did co-localization assays by co-transfecting RFP-tagged *foxo3b* together with GFP-tagged *β-catenin1* or *β-catenin2* into HeLa cells. We observed that *foxo3b* co-localized with *β-catenin1* and *β-catenin2* in the nucleus (Fig. 8B). Then, we performed immunoprecipitation (IP) experiments to further confirm the interaction between *foxo3b* and *β-catenin1/2*. As showed in Figure 8C, *foxo3b* could indeed interact with both *β-catenin1* and *β-catenin2*. The interaction between *foxo3b* and *β-catenin1* seemed to be stronger than that between *foxo3b* and *β-catenin2* (line 4 from left to right in Fig. 8C1 and C2). The protein level of *β-catenin1* was enhanced after co-transfection with *foxo3b* (line 2 from left to right in Fig. 8C1), while the protein level of *β-catenin2* was not changed after co-transfection with *foxo3b* (line 2 from left to right in Fig. 8C2). Taken together, these observations suggested that *foxo3b* interacted with *β-catenin1* and *β-catenin2* to negatively regulate Wnt/β-catenin signaling. The interaction between *foxo3b* and *β-catenin1* was much stronger than that between *foxo3b* and *β-catenin2* implied that *β-catenin1* might act as a major target for *foxo3b* in regulating Wnt/β-catenin signaling during zebrafish early embryogenesis.

## Discussion

Although the function of mammalian FOXO inhibiting β-catenin/T cell factor activity has been revealed through cell-culture system [16], how important of this inhibition *in vivo*, particularly in embryogenesis is still unclear. In this study, we took advantage of zebrafish model thoroughly exploring the role of *foxo3b*, an orthologue of mammalian *FOXO3*, in embryogenesis. We found that zebrafish *foxo3b* played important roles in axis and neuroectoderm formation. Furthermore, we confirmed that *foxo3b* could indeed interact with zebrafish *β-catenin1* and *β-catenin2* to inhibit LEF/TCF-dependent transcription *in vitro* and *in vivo*. In summary, we thought that zebrafish *foxo3b* affected early embryogenesis through negatively regulating maternal and zygotic *β-catenin* transactivity.

### Zebrafish *Foxo3b* Gene is Expressed Maternally and Required for Early Embryogenesis

The opposite roles of zebrafish maternal and zygotic Wnt/β-catenin signaling during embryogenesis have been recognized [4], [7], [8]. In addition, *β-catenin* and TCF activate dorsal-specific genes at the blastula stage, but mediate the activity of the ventro-laterally expressed *wnt8* to repress the dorsal-specific genes during gastrulation [14]. Herein, the major concern was that whether *foxo3b* was expressed maternally or at early stage of embryogenesis. In our study, by *in situ* hybridization, we revealed that high expression of *foxo3b* could be detected in 2-cell stage embryos, implying that *foxo3b* was expressed maternally, and distributed ubiquitously as *β-catenin1*, *β-catenin2* and *ICAT* [4], [41] during the blastula stage (Fig. 1). In addition, semi-quantitative RT-PCR assays also detected *foxo3b* transcripts as early as 2-cell stage, further confirming that *foxo3b* was expressed maternally. Thus, zebrafish *foxo3b* might play important roles during early embryogenesis. Knockdown of *foxo3b* expression by injections of either *foxo3b*-ATG-MO or *foxo3b*-SP-MO, resulted in severe developmental defects including reduced body length, abnormality of brain and eye development, and curved body, which implied that both maternal and zygotic *foxo3b* function importantly in zebrafish body axis formation.

In zebrafish, Wnt/β-catenin signaling has been reported to affect body axis patterning by opposing effect of maternal and zygotic actions [12]. Exaggerated Wnt signaling after the MBT (mid-blastrula transition) by ectopic expression of wnts, β-catenin [12] or by LiCl treating [10], [42], leads to loss of rostral neural domains, whereas reduced Wnt signaling leads to expansion of rostral neural domains. In this study, the defects of anterior brain were observed in both *foxo3b*-ATG-MO morphants and splice-MO morphants, suggesting that these morphants harbored elevated Wnt signaling activity after MBT. Interestingly, the phenotypes exhibited in *foxo3b* morphants were similar to that of the embryos with ectopic expression of *wnts*, *vent*, *vox* and *ved*, all components of zygotic Wnt/β-catenin signaling [6], [7], [8]. As indicated by *cdx4* expansion in *foxo3b* morphants (Fig. 6), *foxo3b* knockdown caused high zygotic wnt8/β-catenin signaling to continually suppress the dorsal organizer and promote the posterior neuroectoderm formation. In addition, the exaggerated zygotic wnt8/β-catenin signaling in the ventro-lateral region disturbed the balance of the opposing actions between dorsal organizer genes *boz*, *gsc* and ventral genes *ved*, *vent* and *vox* in embryos injected with either *foxo3b*-ATG-MO or *foxo3b*-SP-MO, which might cause high rate of anterior neuroectoderm defects (Fig. 2 and Fig. 3). These observations suggested that knockdown of either maternal or zygotic *foxo3b* in embryos resulted in elevated and constant β-catenin signaling in morphants, resulting in anterior brain defects (Fig. 2, Fig. 3 and Fig. 7).

### *Foxo3b* Affects Zebrafish DV Patterning Through Negatively Regulating Both Maternal and Zygotic Wnt/β-catenin Signaling

As reported, the specification of vertebrate body axes, such as the dorsal-ventral (DV) and anterior-posterior (AP) axis, is initiated soon after fertilization. The maternal factors are required to locally activate zygotic genes to specify dorsal-ventral polarity [43], [44] and to induce patterning center (such as Nieuwkoop center and Spemann organizer) formation [45]. Maternal Wnt/β-catenin signaling genes play important roles in this process. In this study, we found that *foxo3b* morphants displayed defects in body axis formation. Further marker gene staining showed that the expression of organizer genes *sqt* and *flh* expanded in *foxo3b* morphants at 30% epiboly (Fig. 3). *Sqt* is a direct target gene of maternal β-catenin [28]. Thus, these results suggested that *foxo3b* might affect the specification of zebrafish body axes through influencing maternal Wnt/β-catenin signaling. In addition, the fact that Wnt target genes *sqt* and *gsc* increased dramatically in *foxo3b* morphants at blastula stage also indicated the up-regulation of Wnt/β-catenin signaling (Fig. 4 and Fig. 7). Furthermore, *gsc* expression could be rescued by co-injection of *β-catenin2-*MO in *foxo3b* morphants at 30% epiboly. However, notably, most co-injected embryos displayed dramatically reduced *gsc* expression (Fig. 7C), which suggested that *β-catenin2*-MO can successfully suppressed the increased expression of *gsc* in *foxo3b* morphants, and *β-catenin2* might function downstream of *foxo3b* in Wnt/β-catenin signaling. Noteworthily, *β-catenin2* morphants but not *β-catenin1* morphants failed to express organizer genes *boz*, *sqt* and *gsc*, which established the importance of maternal *β-catenin2* for organizer formation [4]. Beyond expectation, both *β-catenin2*-MO and *β-catenin1*-MO suppressed the increased expression of organizer genes in *foxo3b* morphants, promoting the possibility that when *β-*catenin1 protein was reduced in *foxo3b* morphants, the remained *foxo3b* protein in morphants could still interact with *β-catenin2* efficiently to continuously suppress the *β-catenin2* activity effectively, resulting in counteraction of the increased expression of *sqt* and *gsc* in *foxo3b* morphants.

The following two observations might support this point. Firstly, *foxo3b* interacted with *β-catenin1* much more efficiently than with *β-catenin2*. Thus, if *β-catenin1* protein level was the same as that of *β-catenin2*, the remained *foxo3b* protein in morphants should be apt to interact with *β-catenin1*. Secondly, low dosage of *foxo3b* mismatch mRNA (125 pg per embryo) could rescue the defects of *foxo3b* morphants efficiently (Fig. 2 and Fig. 3), suggesting that only a little inhibitory effect of foxo3b was required in *foxo3b* morphants to counteract developmental defects.

Moreover, we found the mixed expression of organizer genes *flh* and *gsc* at shield stage in *foxo3b* morphants (Fig. 4B), which suggested that their expression were affected by both the positive influence of maternal Wnt signaling and the negative influence of ventro-lateral zygotic Wnt/β-catenin signaling. These results strongly supported that zebrafish *foxo3b* could inhibit both maternal and zygotic Wnt/β-catenin signaling, similar to that observed for *Naked1/Naked2* genes [31].

Ventralizing transcriptional repressors in the Vox/Vent family have been proposed to serve as important regulators for DV patterning in the early embryogenesis [46], [47], [48], [49]. In addition, BMP gene family is also shown to be an important type of ventral genes [50], [51], [52]. To date, only maternal *runx2* has been identified to induce zygotic *vox, vent* and *ved* expression by directly binding to their promoters [29]. As reported, the zygotic inducers of Vox/Vent family are *wnt8* at 40-50% epiboly and BMP at 75% epiboly. During the developmental stages, dorsal genes, such as *boz* and *gsc* continue to repress *vox, vent* and *ved* expression. In this study, we found that *vox* displayed robust expression at 30% epiboly in *foxo3b* morphants. At this stage, ventro-lateral *wnt8* expression was just initiated, so it was unlikely that zygotic inducer *wnt8* up-regulated *vox* expression. In addition, it was also unlikely that *vox* expression was up-regulated by the organizer, because the expanded organizer was supposed to suppress expression of Vox/Vent family [7]. Thus, the expansion of *vox* expression at 30% epiboly in *foxo3b* morphants might result from directly up-regulation by maternal Wnt/β-catenin signaling. In fact, vox/vent family genes, harboring TCF binding sites in their promoters, have already been shown to be directly regulated by *wnt8* in zebrafish embryos [7]. We further validated this point by rescue experiment that *β-catenin2-*MO could neutralize the increased expression of *vox* in *foxo3b* morphants efficiently (Fig. 7).

In *foxo3b* over-expressed embryos, most embryos displayed expanded *gsc* expression at shield stage, similar to that of embryos over-expressed with *Nkds* [31] or Wnt inhibitors [14], [53]. Over-expression of *Nkds* dramatically reduces the negative influence of zygotic ventro-lateral Wnt/β-catenin signaling on organizer suppression, so *foxo3b* over-expression might also reflect the influence of *foxo3b* on zygotic wnt/β-catenin signaling. In this study, apart from dorsal marker genes staining, we also checked the ventro-lateral wnt target genes expression in *foxo3b* over-expressed embryos. As showed in Figure 5, the results suggested that *foxo3b* might repress β-catenin activity in embryos to cause the down-regulation of vox/vent gene family, which further resulted in promoting dorsal marker gene expression.

### Foxo3b Affects Zebrafish Anterior Neuroectoderm Patterning Through Negatively Regulating Both Maternal and Zygotic Wnt/β-catenin Signaling

Wnt/β-catenin signaling has been identified to define discrete domains of gene expression along the anterior-posterior (AP) axis of the neural plate and then help establish the formation of neural tube compartments along the axis [8], [54], [55]. As reported, *TCF3* (*headless*) is expressed in the anterior neuroectoderm, functioning as a repressor of posterior neural fates in this region [12]. β-catenin signaling activated by *wnt8* participates in posteriorizing the neural plate. Loss of *TCF3* function leads to the over-activity of the Wnt pathway in the anterior part of neuroectoderm and results in defects in anterior fates [12]. Moreover, *bozozok* mutant zebrafish shows defects in anterior neural structures, which can be rescued by dn-X*wnt8* [56]. These previous observations strongly support that exaggerated *wnt8* activity leads to loss of rostral neural domains. In our study, knockdown of *foxo3b* resulted in defects in forebrain and eyes. The expression of forebrain markers in *foxo3b* morphants was relatively reduced (Fig. 3 and Fig. 7). Therefore, in addition to affecting DV patterning, *foxo3b* could also affect forebrain induction through negatively regulating maternal and zygotic wnt/β-catenin signaling. In fact, the reduced expression of forebrain markers in *foxo3b* morphants could be rescued by co-injection of dn*TCF* mRNA (Fig. 7), further verifying that the anterior defects in *foxo3b* morphants might result from exaggerated Wnt/β-catenin activity and *foxo3b* was a negative regulator of wnt/β-catenin signaling.

It has been reported that Wnt/β-catenin signaling antagonizes eye specification through activating *wnt8b* and *fz8a* [57]. Similar results were obtained by studies of *masterblind* (axin mutated) and *headless* zebrafish mutants, which refined the function of wnt/β-catenin signaling in eye formation [12], [58]. In addition, over-expression of *wnt8* in zebrafish results in most embryos failing to develop one or both eyes [8]. In *foxo3b* morphants, eye formation was disrupted to different extent, as indicated by reduction of *tbx5*, *six3b* and *pax6* expression in retina. These results further highlighted the role of *foxo3b* in maintenance of anterior neural fates.

## Supporting Information

Figure S1 *Bmp2b* expression was rescued in *foxo3b*-knockdown embryos co-injected with *foxo3b* mismatch mRNA. Animal views, shield stage.
(TIF)

Table S1 The partial primers used for cloning probes.
(DOC)

## References

[1] Huang H, Tindall DJ (2007). Dynamic FoxO transcription factors.. J Cell Sci.

[2] Carter ME, Brunet A (2007). FOXO transcription factors.. Curr Biol.

[3] Hosaka T, Biggs WH, Tieu D, Boyer AD, Varki NM (2004). Disruption of forkhead transcription factor (FOXO) family members in mice reveals their functional diversification.. Proc Natl Acad Sci U S A.

[4] Bellipanni G, Varga MT, Maegawa S, Imai Y, Kelly C (2006). Essential and opposing roles of zebrafish beta-catenins in the formation of dorsal axial structures and neurectoderm.. Development.

[5] Lekven AC, Thorpe CJ, Waxman JS, Moon RT (2001). Zebrafish wnt8 encodes two wnt8 proteins on a bicistronic transcript and is required for mesoderm and neurectoderm patterning.. Dev Cell.

[6] Shimizu T, Yamanaka Y, Nojima H, Yabe T, Hibi M (2002). A novel repressor-type homeobox gene, ved, is involved in dharma/bozozok-mediated dorsal organizer formation in zebrafish.. Mechanisms of Development.

[7] Ramel MC, Lekven AC (2004). Repression of the vertebrate organizer by Wnt8 is mediated by Vent and Vox.. Development.

[8] Kelly GM, Greenstein P, Erezyilmaz DF, Moon RT (1995). Zebrafish wnt8 and wnt8b share a common activity but are involved in distinct developmental pathways.. Development.

[9] Bouwmeester T, Kim S, Sasai Y, Lu B, De Robertis EM (1996). Cerberus is a head-inducing secreted factor expressed in the anterior endoderm of Spemann's organizer.. Nature.

[10] Leyns L, Bouwmeester T, Kim SH, Piccolo S, De Robertis EM (1997). Frzb-1 is a secreted antagonist of Wnt signaling expressed in the Spemann organizer.. Cell.

[11] Glinka A, Wu W, Delius H, Monaghan AP, Blumenstock C (1998). Dickkopf-1 is a member of a new family of secreted proteins and functions in head induction.. Nature.

[12] Kim CH, Oda T, Itoh M, Jiang D, Artinger KB (2000). Repressor activity of Headless/Tcf3 is essential for vertebrate head formation.. Nature.

[13] Dorsky RI, Itoh M, Moon RT, Chitnis A (2003). Two tcf3 genes cooperate to pattern the zebrafish brain.. Development.

[14] Pelegri F, Maischein HM (1998). Function of zebrafish beta-catenin and TCF-3 in dorsoventral patterning.. Mech Dev.

[15] Essers MA, de Vries-Smits LM, Barker N, Polderman PE, Burgering BM (2005). Functional interaction between beta-catenin and FOXO in oxidative stress signaling.. Science.

[16] Hoogeboom D, Essers MA, Polderman PE, Voets E, Smits LM (2008). Interaction of FOXO with beta-catenin inhibits beta-catenin/T cell factor activity.. J Biol Chem.

[17] Biggs WH, 3rd, Cavenee WK, Arden KC (2001). Identification and characterization of members of the FKHR (FOX O) subclass of winged-helix transcription factors in the mouse.. Mamm Genome.

[18] Liu JX, Hu B, Wang Y, Gui JF, Xiao W (2009). Zebrafish eaf1 and eaf2/u19 mediate effective convergence and extension movements through the maintenance of wnt11 and wnt5 expression.. J Biol Chem.

[19] Westerfield M, Doerry E, Douglas S (1999). Zebrafish in the Net.. Trends Genet.

[20] Kimmel CB, Ballard WW, Kimmel SR, Ullmann B, Schilling TF (1995). Stages of embryonic development of the zebrafish.. Dev Dyn.

[21] Yang Z, Liu N, Lin S (2001). A zebrafish forebrain-specific zinc finger gene can induce ectopic dlx2 and dlx6 expression.. Dev Biol.

[22] Zhou J, Feng X, Ban B, Liu J, Wang Z (2009). Elongation factor ELL (Eleven-Nineteen Lysine-rich Leukemia) acts as a transcription factor for direct thrombospondin-1 regulation.. J Biol Chem.

[23] Begemann G, Ingham PW (2000). Developmental regulation of Tbx5 in zebrafish embryogenesis.. Mech Dev.

[24] Erter CE, Wilm TP, Basler N, Wright CVE, Solnica-Krezel L (2001). Wnt8 is required in lateral mesendodermal precursors for neural posteriorization in vivo.. Development.

[25] Szeto DP, Kimelman D (2004). Combinatorial gene regulation by Bmp and Wnt in zebrafish posterior mesoderm formation (vol 131, pg 3751, 2004).. Development.

[26] Ramel MC, Buckles GR, Baker KD, Lekven AC (2005). WNT8 and BMP2B co-regulate non-axial mesoderm patterning during zebrafish gastrulation.. Dev Biol.

[27] Kelly C, Chin AJ, Leatherman JL, Kozlowski DJ, Weinberg ES (2000). Maternally controlled (beta)-catenin-mediated signaling is required for organizer formation in the zebrafish.. Development.

[28] Shimizu T, Yamanaka Y, Ryu SL, Hashimoto H, Yabe T (2000). Cooperative roles of Bozozok/Dharma and Nodal-related proteins in the formation of the dorsal organizer in zebrafish.. Mech Dev.

[29] Flores MV, Lam EY, Crosier KE, Crosier PS (2008). Osteogenic transcription factor Runx2 is a maternal determinant of dorsoventral patterning in zebrafish.. Nat Cell Biol.

[30] Hoppler S, Brown JD, Moon RT (1996). Expression of a dominant-negative Wnt blocks induction of MyoD in Xenopus embryos.. Genes Dev.

[31] Van Raay TJ, Coffey RJ, Solnica-Krezel L (2007). Zebrafish Naked1 and Naked2 antagonize both canonical and non-canonical Wnt signaling.. Dev Biol.

[32] Brunet A, Bonni A, Zigmond MJ, Lin MZ, Juo P (1999). Akt promotes cell survival by phosphorylating and inhibiting a Forkhead transcription factor.. Cell.

[33] Murphy CT, McCarroll SA, Bargmann CI, Fraser A, Kamath RS (2003). Genes that act downstream of DAF-16 to influence the lifespan of Caenorhabditis elegans.. Nature.

[34] Christian M, Zhang XH, Schneider-Merck T, Unterman TG, Gellersen B (2002). Cyclic AMP-induced forkhead transcription factor, FKHR, cooperates with CCAAT/enhancer-binding protein beta in differentiating human endometrial stromal cells.. Journal of Biological Chemistry.

[35] Shimizu T, Bae YK, Muraoka O, Hibi M (2005). Interaction of Wnt and caudal-related genes in zebrafish posterior body formation.. Dev Biol.

[36] Seiliez T, Thisse B, Thisse C (2006). FoxA3 and goosecoid promote anterior neural fate through inhibition of Wnt8a activity before the onset of gastrulation.. Developmental Biology.

[37] Wilm TP, Solnica-Krezel L (2005). Essential roles of a zebrafish prdm1/blimp1 homolog in embryo patterning and organogenesis.. Development.

[38] Martin BL, Kimelman D (2008). Regulation of canonical Wnt signaling by Brachury is essential for posterior mesoderm formation.. Developmental Biology.

[39] Seoane J, Le HV, Shen LJ, Anderson SA, Massague J (2004). Integration of Smad and Forkhead pathways in the control of neuroepithelial and glioblastoma cell proliferation.. Cell.

[40] Stahl M, Dijkers PF, Kops GJPL, Lens SMA, Coffer PJ (2002). The forkhead transcription factor FoxO regulates transcription of p27(Kip1) and bim in response to IL-2.. Journal of Immunology.

[41] Tago K, Nakamura T, Nishita M, Hyodo J, Nagai S (2000). Inhibition of Wnt signaling by ICAT, a novel beta-catenin-interacting protein.. Genes Dev.

[42] Kim SH, Shin J, Park HC, Yeo SY, Hong SK (2002). Specification of an anterior neuroectoderm patterning by Frizzled8a-mediated Wnt8b signalling during late gastrulation in zebrafish.. Development.

[43] Steward R, Govind S (1993). Dorsal-ventral polarity in the Drosophila embryo.. Curr Opin Genet Dev.

[44] Reim G, Brand M (2006). Maternal control of vertebrate dorsoventral axis formation and epiboly by the POU domain protein Spg/Pou2/Oct4.. Development.

[45] Moon RT, Kimelman D (1998). From cortical rotation to organizer gene expression: toward a molecular explanation of axis specification in Xenopus.. Bioessays.

[46] Imai Y, Gates MA, Melby AE, Kimelman D, Schier AF (2001). The homeobox genes vox and vent are redundant repressors of dorsal fates in zebrafish.. Development.

[47] Gawantka V, Delius H, Hirschfeld K, Blumenstock C, Niehrs C (1995). Antagonizing the Spemann organizer: role of the homeobox gene Xvent-1.. EMBO J.

[48] Onichtchouk D, Gawantka V, Dosch R, Delius H, Hirschfeld K (1996). The Xvent-2 homeobox gene is part of the BMP-4 signalling pathway controlling [correction of controling] dorsoventral patterning of Xenopus mesoderm.. Development.

[49] Onichtchouk D, Glinka A, Niehrs C (1998). Requirement for Xvent-1 and Xvent-2 gene function in dorsoventral patterning of Xenopus mesoderm.. Development.

[50] Kishimoto Y, Lee KH, Zon L, Hammerschmidt M, Schulte-Merker S (1997). The molecular nature of zebrafish swirl: BMP2 function is essential during early dorsoventral patterning.. Development.

[51] Dosch R, Gawantka V, Delius H, Blumenstock C, Niehrs C (1997). Bmp-4 acts as a morphogen in dorsoventral mesoderm patterning in Xenopus.. Development.

[52] Hoppler S, Moon RT (1998). BMP-2/-4 and Wnt-8 cooperatively pattern the Xenopus mesoderm.. Mech Dev.

[53] Kawahara A, Wilm T, Solnica-Krezel L, Dawid IB (2000). Antagonistic role of vega1 and bozozok/dharma homeobox genes in organizer formation.. Proc Natl Acad Sci U S A.

[54] Kelly GM, Moon RT (1995). Involvement of wnt1 and pax2 in the formation of the midbrain-hindbrain boundary in the zebrafish gastrula.. Dev Genet.

[55] Keynes R, Lumsden A (1990). Segmentation and the origin of regional diversity in the vertebrate central nervous system.. Neuron.

[56] Fekany-Lee K, Gonzalez E, Miller-Bertoglio V, Solnica-Krezel L (2000). The homeobox gene bozozok promotes anterior neuroectoderm formation in zebrafish through negative regulation of BMP2/4 and Wnt pathways.. Development.

[57] Cavodeassi F, Carreira-Barbosa F, Young RM, Concha ML, Allende ML (2005). Early stages of zebrafish eye formation require the coordinated activity of Wnt11, Fz5, and the Wnt/beta-catenin pathway.. Neuron.

[58] Heisenberg CP, Houart C, Take-Uchi M, Rauch GJ, Young N (2001). A mutation in the Gsk3-binding domain of zebrafish Masterblind/Axin1 leads to a fate transformation of telencephalon and eyes to diencephalon.. Genes Dev.

