# Supplementary figures and images for: Zebrafish foxo3b Negatively Regulates Canonical Wnt Signaling to Affect Early Embryogenesis
Source: PLoS One. 2011 Sep 7;6(9):e24469. doi: 10.1371/journal.pone.0024469 (PMC3168510; doi:10.1371/journal.pone.0024469)

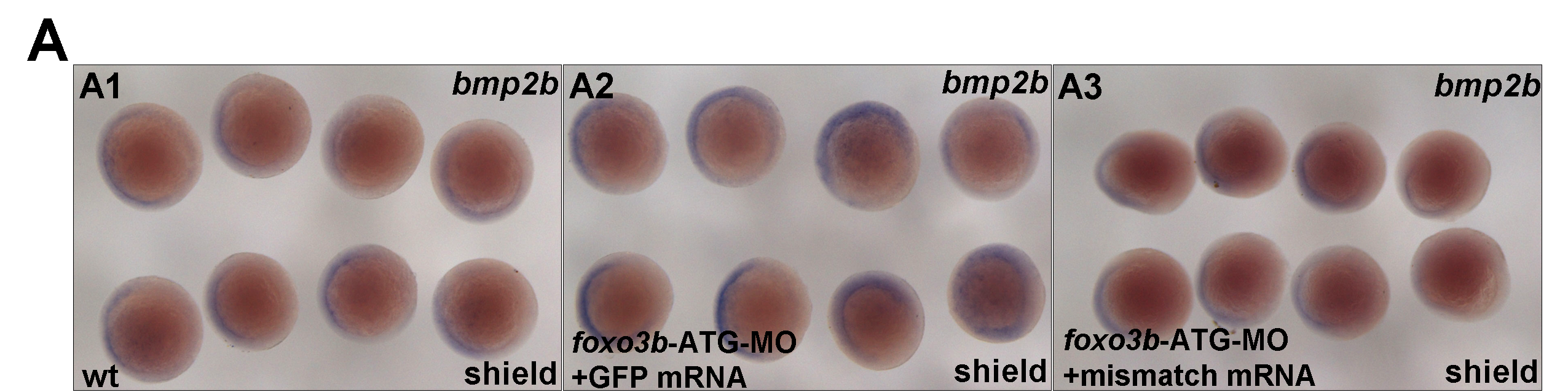

Supplement: Figure S1 — Bmp2b expression was rescued in foxo3b-knockdown embryos co-injected with foxo3b mismatch mRNA. Animal views, shield stage. (TIF) [file pone.0024469.s001.tif]
