# Supplementary material for: Zebrafish foxo3b Negatively Regulates Canonical Wnt Signaling to Affect Early Embryogenesis
Source: PLoS One. 2011 Sep 7;6(9):e24469. doi: 10.1371/journal.pone.0024469 (PMC3168510; doi:10.1371/journal.pone.0024469)
Supplement: Table S1 — The partial primers used for cloning probes. (DOC) [file pone.0024469.s002.doc]

| **Supplemental Table 1. The partial primers used for probe cloning** | |
| --- | --- |
| *opl* | Opl-F, GGCGAAGTTACAGACAGA;  Opl-R, GACATGACCGTATTGCTC; |
| *six3b* | Six3b-F, TTTGGTCGTTGCCCGTAG;  Six3b-R, CGTGATGCTGAAG CCTGT. |
| *foxo3a* | Foxo3a-F, TCCAGAACCAAACGAACCTC  Foxo3a-R, TCCGTCCATCAAACCAGC |
| *foxo3b* | Foxo3b-F, CTGTTAGTCTGAATCCTGTGG  Foxo3b-R, TTTCGTTATAGTCTGGGTCTGC |
| *foxi1* | Foxi1-F, TCAGCGTCAACAATCTCAT  Foxi1-R, GTTATAGTGGCCTAGATGTATT |
| *pax6* | Pax6-F, TACTGGCCCAGACTACAGTGAG  Pax6-R, GATGAGTCATCAATATGGTCTACTG |
| *cdx4* | cdx4-F,CGTCCATGAGGAACATACAGC  Cdx4-R,CAAGAGCCTCCAGCATTTCG |
